# A case report of hemolytic streptococcal gangrene in the danger triangle of the face with thrombocytopenia and hepatitis

**DOI:** 10.1186/s12887-018-1177-9

**Published:** 2018-06-22

**Authors:** Xiao-ling Jia, Janak L. Pathak, Jin-fa Tong, Ji-mei Su

**Affiliations:** 1 Department of Stomatology, Hangzhou Hospital of Traditional Chinese Medicine, Hangzhou, 310007 Zhejiang China; 2 Key Laboratory of Oral Medicine, Guangzhou Institute of Oral Disease, Stomatological Hospital of Guangzhou Medical University, Guangzhou, 510140 China; 3 Department of Stomatology, Children’s Hospital, Zhejiang University School of Medicine, NO.3333 Binsheng Road, Hangzhou, 310052 Zhejiang Province People’s Republic of China

**Keywords:** Hemolytic *streptococcus* gangrene, Group-a beta-hemolytic *streptococcus*, The danger triangle of the face, Thrombocytopenia, Hepatitis

## Abstract

**Background:**

Hemolytic *streptococcus* gangrene is a life threatening invasive bacterial infection. Hemolytic *streptococcus* gangrene in the danger triangle of the face is too lethal to operate. A case of the confirmed hemolytic *streptococcus* gangrene in the danger triangle of the face caused by Group A beta-hemolytic *streptococcus* (GAS) in 20-months old boy is presented to draw attention of clinicians to this uncommon but frequently fatal infection.

**Case presentation:**

Previously healthy 20 months old boy suddenly developed paranasal gangrene on the left side of the danger triangle of the face, followed by rapidly progressive thrombocytopenia and hepatitis. The clinical features, liver function, and hematological and serological parameters resembled to a description of streptococcal toxic shock syndrome (STSS). Aggressive antibiotics, substitutional and supportive therapy were conducted without surgical debridement of facial tissues. Prompt diagnosis and aggressive timely treatment completely cured the disease in 28 days.

**Conclusions:**

The present case report demonstrates prompt diagnosis and timely treatment as a strategy to cure the fatal hemolytic *streptococcus* gangrene located in too risky body part to operate.

## Background

Hemolytic *streptococcus* gangrene is invasive bacterial infection mainly caused by GAS. Human are the natural hosts and sole reservoirs for GAS. Necrotizing soft tissue infections (NSTI) are among the serious consequences caused by GAS infection. GAS-caused NSTI are characterized by frequent development of shock and high mortality [1]. GAS infection-related in-hospital case fatality rate is reported to be about 11% [1]. The incidence of the GAS infection has been reported to increase during the last 10–20 years due to the increasing colonization of the GAS in general population [2]. Hemolytic *streptococcus* gangrene is a fatal disease that causes systemic illness and multisystem failures, such as bacteremia, renal impairment, hepatitis, acute thrombocytopenia and respiratory failure. Hemolytic *streptococcus* gangrene in the danger triangle of the face is exceedingly lethal and rare. Early diagnosis, aggressive timely treatment and prompt initiation of supportive care are crucial for a good prognosis. We reported a case of early diagnosis and successful treatment of hemolytic *streptococcus* gangrene in a 20-month-old boy, who developed severe hemolytic *streptococcus* gangrene in the danger triangle of the face followed by rapidly progressive thrombocytopenia and hepatitis. We diagnosed hemolytic streptococcus gangrene based on the clinical symptoms, signs of the disease, bacterial isolation and identification, hematological markers, serological markers for vital organ test, and B-ultrasonography of liver and spleen.

## Case presentations

**Fig. 1:**
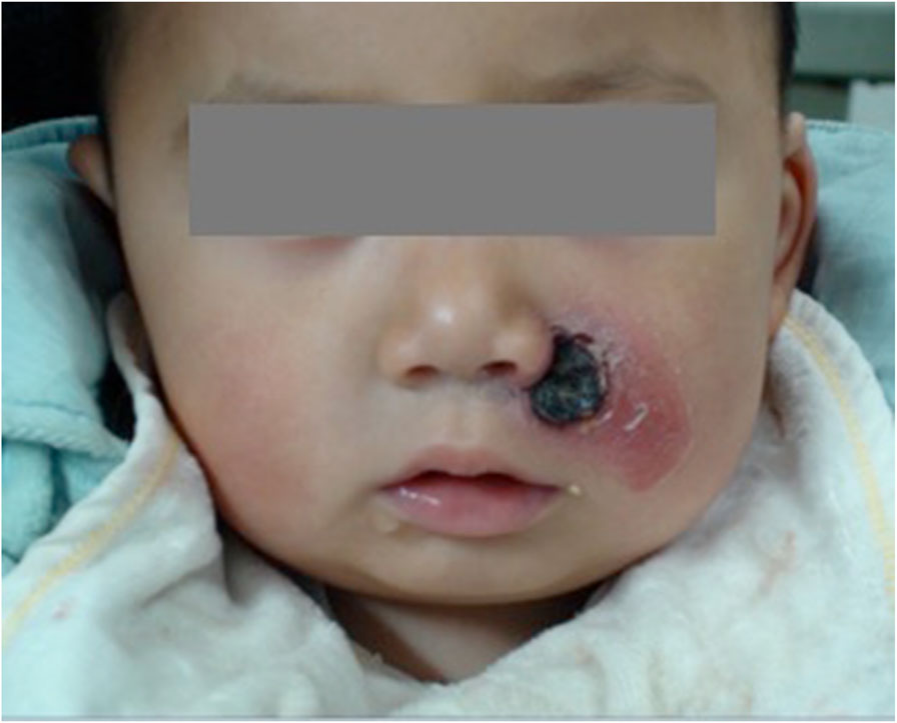
Image of hemolytic *streptococcus* gangrene in the left maxillofacial region upon admission

A 20-month-old boy was referred to our hospital due to paranasal gangrene on the left side of the maxillofacial danger triangle (Fig. 1). The boy presented with a flu-like syndrome with fever, cough, shivering and sore throat four days prior to referral. Redness, swelling and pain occurred along with several vesicles, and a bloody secretion appeared in his left paranasal region two days prior to referral. The boy was given intensive intravenous penicillin G therapy for four days at the local hospital but there was no sign of regression. Swelling in left paranasal region worsened two days prior to referral. The medical history showed that the boy was born after an uneventful pregnancy, and had a normal growth and development. He had no history of medical illness, including diabetes mellitus or cardiac conditions. No history suggested that trauma, skin abrasions, insect bites or sinusitis had occurred.

Upon admission to our hospital, the patient was conscious and stable but very weak with body temperature of 103.5 °F, a respiratory rate of 32/min, a heart rate of 146/min, and blood pressure of 108/69 mmHg. Steady breathing with slightly red throat was observed. Rough breathing sounds were heard in the both lungs with no rales. The cardiac auscultation revealed a regular rate and rhythm. There was no sign of abdominal tenderness and neurological abnormalities.

A facial examination showed a red, swollen substantially infected area (approximately 4 × 3 cm) that involved the left nasolabial groove, left cheek and left upper lip (Fig. 1). A gangrenous region (approximately 1.5 × 1.5 cm) was found in the left paranasal maxillofacial danger triangle with no purulent secretion (Fig. 1). The gangrene extended into the subcutaneous fat tissue but did not involve the fascia and muscles. The demarcation from areas of necrosis to more normal tissue was nearly clear. The intraoral mucosa was not red or swollen (Fig. 1).

**Fig. 2:**
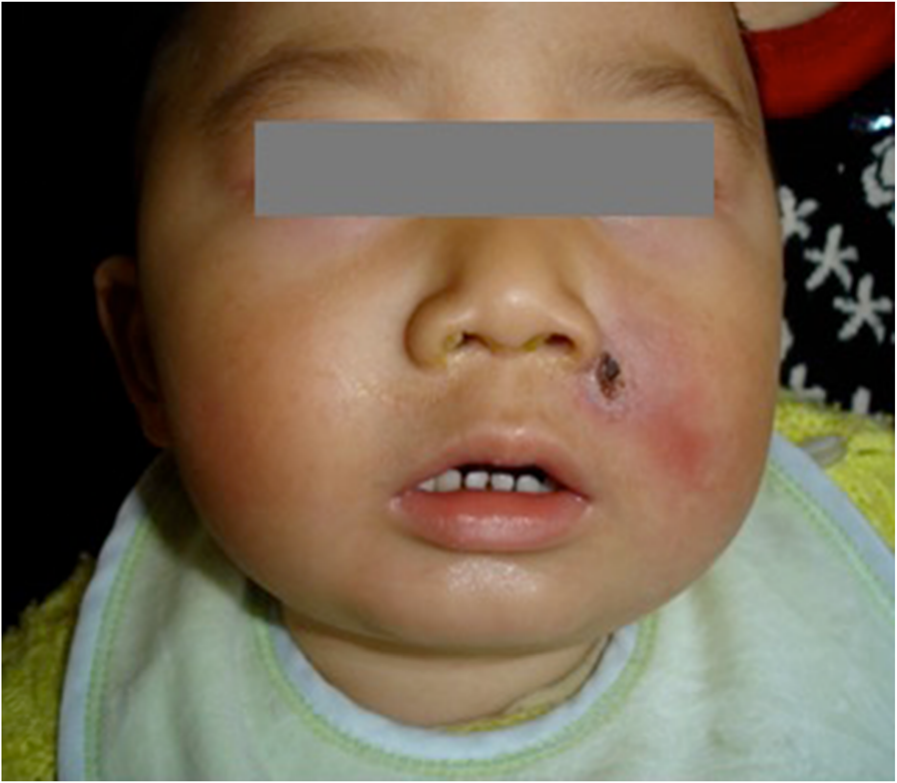
Image of hemolytic *streptococcus* gangrene in the left maxillofacial region after 7 days of admission

A rapid laboratory examination showed a significantly decreased platelet count of 48 × 10^9^/L, a reduced hemoglobin (HB) concentration associated with an elevated erythrocyte sedimentation rate (ESR) of 56 mm/h, and a C-reactive protein (CRP) level of 160 mg/L. Local changes worsened after four days of intravenous penicillin G (800 thousand unit, twice a day) therapy, and the epithelial defect was more prominent with worsening hematoma. Clinical features and laboratory data indicated this case as a more serious illness than initially thought. Empiric antibiotic therapy with intravenous vancomycin (0.15 g every 8 h for 15 days) and meropenem (0.15 g every 8 h for 9 days) was started immediately to control the facial tissue infection. Surgical debridement of facial tissue was not performed due to risky location of the infection i.e. the danger triangle of the face. Surgery in the danger triangle of the face poses a high risk of intracranial infection, which is often fatal. After 24 h of patient admission, pus culture report showed heavy growth of GAS along with a small growth of *Staphylococcus aureus*. Antibacterial sensitivity test showed that both GAS and *Staphylococcus aureus* were sensitive to vancomycin and meropenem but resistant to penicillin G. After 3 days of treatment, body temperature returned to normal, and the facial infection was controlled. However, the platelet count continued to decrease. Support measures were applied immediately, including intravenous gamma globulin (10 g per day) and hexadecadrol (1 mg per day) until the platelet count recovered to a normal level. On the 8th day, liver function markers were significantly elevated. Elevated serum glutamic-pyruvic transaminase (GPT, 1645 U/L), glutamic oxaloacetic transaminase (GOT, 866 U/L) and gamma-glutamyl transpeptidase (GGT, 182 U/L) were observed (Table 1). B-ultrasonography revealed hepatosplenomegaly. The patient was treated with hepatinica combined with nutritional supportive therapy. The gangrene in the maxillofacial region began to subside after 7 days (Fig. 2). On the 15th day, the hepatic function was substantially improved.

**Table 1:** Laboratory parameters of a patient with hemolytic *streptococcus* gangrene

Reference value	day 1	day 2	day 5	day 8	day 11	day 15	day 21	day 28
PLT:100–400 *10^9^/L	48	37	12	106	173	177	212	*268*
HB:110–155 g/L	79	75	85	86	87	91	105	*119*
ESR:0–20 mm/h		56	122	124	111	119	78	*64*
CRP: < 1 mg/L	> 160		44	14	< 1	< 1		*< 1*
GPT:5–50 U/L	64			1645	642	183	90	*109*
*GOT:*5–55 U/L	35			866	142	48	93	*79*
*GGT:5–50 U/L*	*15*			*182*	*107*	*57*	*38*	*29*

PLT-Platelet count; ESR-Erythrocyte sedimentation rate; CRP-C-reactive protein

GPT-Glutamic-pyruvic transaminase; GOT-Glutamic oxaloacetic transaminase

GGT-Gamma-glutamyl transpeptidase

This patient was therefore diagnosed with hemolytic *streptococcal* gangrene, thrombocytopenia and hepatitis. Early diagnosis and aggressive timely treatment cured the infection within 28 days.

## Discussion and conclusions

GAS causes about 500,000 deaths every year in the world [3]. GAS possesses considerable extracellular virulence factors to cause infection. These virulence factors are associated with bacterial adhesion and spreading, tissue destruction, immune system evasion, and cellular toxicity [4]. In around 10% of GAS cases, superantigen toxins produced by the bacteria stimulate a large proportion of T cells leading to STSS [5]. The pathogen spreads through droplets from parts of an infected tissue [6]. In this case report, patient developed a flu-like syndrome prior to the maxillofacial infection.

The pathogenicity of GAS ranges from mild infections such as impetigo or pharyngitis to severe invasive infections such as hemolytic *streptococcus* gangrene, necrotizing fasciitis or STSS. Clinical characteristics of GAS are often markedly different in individuals infected with the same strain. Such difference is resulted from a complex interaction between the bacterial virulence factors, the mode of infection and the individual host immunity [7]. GAS genotype emm1 (range 20–33%) is the leading cause of invasive disease worldwide followed by emm28 (with a range of 15%). Both genotypes are susceptible to penicillin [8]. However, emm3 genotype had also been reported to be more commonly associated with death than other emm genotypes [9]. Therefore, the genotype related severity of GAS is still a controversy.

Old age, cardiopulmonary or hepatorenal diseases, diabetes mellitus, debility, obesity, peripheral arteriovenous malformation or lymphatic insufficiency, and trauma are among the factors associated with the risk of death during invasive *streptococcal* infection [10–12]. Necrotizing fasciitis carries the highest risk of GAS-related mortality. However necrotizing fasciitis is a relatively rare condition that accounts for approximately 10% of GAS-related deaths [13]. The second most important prognostic factor is the presence of STSS. The mortality rate in patients without STSS and with STSS had been reported to be approximately 30% and 80–100% respectively [14]. The time of diagnosis is a very crucial prognostic factor. Distinguishing between simple cellulitis and hemolytic *streptococcus* gangrene during early course of the infection is a difficult task. A complete blood count, CRP level, and liver and kidney function tests should be ordered for patients with severe infections and comorbidities causing organ dysfunction on admission. Blood cultures are useful in patients with signs of severe and systemic infections [15]. Tissue biopsies are the preferred diagnostic test for necrotizing skin and soft tissue infections [16]. Imaging techniques provide extra evidences for diagnosis of GAS. Clinical examination combined with imaging studies increases the accuracy of diagnosis and the depth of the infection [17]. In this study, early speculation and diagnosis of GAS was a life saving event.

Meleney reported 20 cases of *streptococcal* gangrene in 1924. He listed extensive gangrene as an essential component of the clinical syndrome [18]. Initial symptoms and signs of hemolytic *streptococcus* gangrene are often similar to acute thrombophlebitis, acute arthritis, acute vascular occlusion or deep soft-tissue trauma. Approximately 130 cases of GAS with 11 cases involving head and neck have been reported in the literature so far [19]. In our case, hemolytic *streptococcus* gangrene in facial area was followed by rapidly progressive thrombocytopenia and toxic hepatitis. These clinical features and multisystem effects were similar to a description of STSS [20]. In STSS, only bacterial culture reports distinguish between *streptococcal* and *staphylococcal* infection. Therefore, antibacterial choice must include coverage of both *streptococcus* and *staphylococcus*. In addition, early administration of intravenous immunoglobulin therapy should be considered in cases of STSS and hemolytic *streptococcus* gangrene [5]. In our case, both GAS and *staphylococcal aureus* were sensitive to both empiric antibiotics vancomycin and meropenem. We also administrated intravenous gamma globulin and methylprednisolone until the multiple organ function and coagulation disorders were improved.

The basic principle of management of acute hemolytic *streptococcus* gangrene has not changed from the approaches advocated by Meleney. Meleney recommended prompt diagnosis, empiric polymicrobial antibiotic therapy, inpatient treatment, surgical removal of debridement and nutritional supportive measures to treat GAS [21]. Since GAS is usually sensitive to penicillin, erythromycin, cephalexin, cloxacillin, vancomycin, minocycline or ciprofloxacin [22], penicillin is still the first choice of treatment. However, cases with failure of penicillin to eradicate GAS from GAS carriers are increasing [23]. Moreover, the presence of *staphylococci* and gram-negative anaerobes during GAS infection suggests for the broad spectrum antibacterial therapy instead of penicillin [24]. In our case, four days of intravenous penicillin G in the local hospital produced no signs of regression. Then established and wide-spectrum antibiotics, such as vancomycin and meropenem, were given intravenously every 8 h. Meropenem could cross the blood-brain barrier to prevent intracranial infection of gangrene in the paranasal maxillofacial danger triangle. We did not perform debridement of necrotic tissue, which would cause severe maxillofacial deformities. We hypothesized that the reasons that the gangrene subsided in this case report were as follows: (a) loose maxillofacial soft tissues with a rich blood supply ensured a strong anti-infection ability, (b) the age of the patient with a strong body metabolism helped to heal the wound, (c) established, sensitive and wide-spectrum antibiotics controlled the infection, (d) prompt diagnosis and aggressive management alleviated the clinical complications and (e) the gangrene did not extend into the fascia and muscles.

This is the first case report of hemolytic *streptococcus* gangrene in the danger triangle of the face of pediatric patient. The present case report demonstrates that prompt diagnosis and timely treatment could treat the fatal hemolytic *streptococcus* gangrene and save patients’ life.

## Abbreviations

CRP: C-reactive protein
ESR: Erythrocytes sedimentation rate
GAS: Group A beta-hemolytic *streptococcus*
GOT: Glutamic oxaloacetic transaminase
GPT: Glutamic-pyruvic
GTT: Gamma-glutamyl transpeptidase
Hb: Hemoglobin
NSTI: Necrotizing soft tissue infections
STSS: *Streptococcal* toxic shock syndrome
